# 

**Published:** 1897-06

**Authors:** 


### CONTENTS:

SELECTIONS.

Article I.—The Reason of the Faith that is in Me. By Linaeus C. Shecut.......... 49
" II.—Two Interesting Cases of Capped Pulps. By R. E. Sparks, M. D., D. D. S.......... 52
" III.—Pulpless Teeth Again—Repeated Dressings. By J. W. Wassall.......... 54
" IV.—Cocaine. By Dr. W. I. Jones.......... 57
" V.—Cleaning the Teeth. By Dr. J. C. Wing.......... 59
" VI.—Compound Cement and Amalgam Filling. By Dr H. Baldwin.......... 60
" VII.—Oral Surgery: Theory and Results. By G. Lenox Curtis, M. D.......... 63
" VIII.—Incidents in Office Practice. By G. V. N. Relyea, L.D.S. 76
" IX.—A Professional Warning. By Mark G. M'Elhinney, D. D. S., L. D. S.......... 77
" X.—The Care of the Teeth. By F. H. Funston, M. D.... 83

COMMUNICATED.

The National Association of Dental Examiners.......... 84
American Dental Association.......... 85

EDITORIAL, ETC.

Impressions of Modeling Compound.......... 85

BIBLIOGRAPHICAL.

Transactions of the New York Odontological Society for 1895.......... 86
Dental Chemistry and Metallurgy.......... 86

MONTHLY SUMMARY.

Dental Operations During Pregnancy.......... 87

Swaging Plates.......... 87

A Light Artificial Denture.......... 88

Space in Filling.......... 89

Absorbed Ridge.......... 89

An Impression and Its Tests.......... 90

Eucaine.......... 91

Carbolate of Iodin.......... 92

Galvanic Action in the Mouth.......... 92

Difficult Partial Impressions.......... 93

To Prevent Rust.......... 93

Function of the Upper Third Molar.......... 94

To Diagnose Pulpitis.......... 95

To Relieve Sensitiveness After Grinding a Tooth.......... 96

